# Immune-Mediated Platelet Activation in COVID-19 and Vaccine-Induced Immune Thrombotic Thrombocytopenia

**DOI:** 10.3389/fimmu.2022.837629

**Published:** 2022-02-22

**Authors:** Günalp Uzun, Lisann Pelzl, Anurag Singh, Tamam Bakchoul

**Affiliations:** ^1^ Center for Clinical Transfusion Medicine, University Hospital of Tuebingen, Tuebingen, Germany; ^2^ Institute of Clinical and Experimental Transfusion Medicine, University Hospital of Tuebingen, Tuebingen, Germany

**Keywords:** thrombosis, platelet activation, coagulation, procoagulant platelets, COVID-19

## Abstract

Both qualitative and quantitative platelet abnormalities are common in patients with coronavirus disease 2019 (COVID-19) and they correlate with clinical severity and mortality. Activated platelets contribute to the prothrombotic state in COVID-19 patients. Several groups have shown immune-mediated activation of platelets in critically ill COVID-19 patients. Vaccine-induced immune thrombotic thrombocytopenia is an autoimmune condition characterized by thrombocytopenia and life-threatening thrombotic events in the arterial and venous circulation. Although the initial trigger has yet to be determined, activation of platelets by immune complexes through Fc gamma RIIA results in platelet consumption and thrombosis. A better understanding of platelet activation in COVID-19 as well as in vaccine-induced thrombotic complications will have therapeutic implications. In this review, we focused on the role of immune-mediated platelet activation in thrombotic complications during COVID-19 infection and vaccine-induced immune thrombotic thrombocytopenia.

## Introduction

Almost two years have passed since the identification of the first cases of new coronavirus-related pneumonia in Wuhan, China (1). Worldwide more than 250 million people have been infected with severe acute respiratory syndrome coronavirus type 2 (SARS-CoV-2), of whom 5.2 million have died (2). COVID-19 starts as an upper respiratory tract infection and progresses to acute respiratory distress syndrome and multiorgan failure in critical cases. Thrombocytopenia is a common laboratory finding in COVID-19 patients (3). The correlation between thrombocytopenia and high mortality suggests that platelets might play an important role in the pathophysiology of COVID-19 (4, 5). In addition, several studies have shown that severe COVID-19 is associated with abnormalities in platelet morphology and function (6, 7). However, it is not clear, how platelets are activated in COVID-19 patients. IgG from COVID-19 patients induces platelet activation in an Fc-gamma-RIIA receptor (FcγRIIA) dependent manner (8–11). SARS-CoV-2 can activate platelets and result in programmed cell death and extracellular vesicle release (12). These findings together indicate that platelets initiate a rapid response to SARS-CoV-2 leading to significant changes in their functional status, which in turn can contribute to dysregulated immunity and thrombosis.

To contain the COVID 19 pandemic, enormous efforts were made to develop effective vaccines in a historically short time. Regulatory authorities have approved a number of COVID-19 vaccines following an accelerated assessment process but implemented a rigorous postmarketing surveillance program for vaccine safety. To date, more than 7 billion doses of vaccine have been administered worldwide, which has shown mainly positive results (2). However, reports of fatal cases with thrombocytopenia and thrombosis of cerebral venous sinuses after vaccination have received increased attention shortly after the rollout of vector-based vaccines in Europe and the USA (13–15). A new syndrome, vaccine-induced immune thrombotic thrombocytopenia (VITT), has been defined after vaccination with the ChAdOx01 nCoV-19 (Astra-Zeneca) and Ad.26.COV2.S (Johnson & Johnson) vaccines (13, 14, 16–18). The exact pathophysiology of VITT remains unclear. Early evidence suggests immune-mediated platelet activation as the cause of thrombotic complications and platelet clearance in VITT (13, 16).

In this review, we summarize the current evidence on the role of immune-mediated platelet activation in thrombotic complications of COVID-19 and VITT.

## Thrombosis in COVID-19

Both venous and arterial thromboembolic events are common in patients with COVID-19 (19, 20). The incidence of pulmonary embolism is 10.5% in patients admitted to a ward and 24.7% in patients admitted to the intensive care unit (21). The adjusted cumulative risks of venous thromboembolism and arterial thrombosis are 23% and 3.1% after 30 days in the intensive care unit, respectively (22).

The pathophysiology of COVID-19-associated coagulopathy includes activation of the coagulation system, endothelial injury, inhibition of fibrinolysis, and release of prothrombotic mediators by immune cells (23). Activated platelets contribute to the prothrombotic state in COVID-19 patients. However, the mechanism of platelet activation in COVID-19 patients is not fully understood.

### Platelet Abnormalities in COVID-19

Critically ill COVID-19 patients develop thrombocytopenia (3). Increased platelet consumption or reduced production of new platelets in the bone marrow are proposed to explain thrombocytopenia (24, 25). In addition to platelet count, alterations in platelet morphology were also observed in COVID-19 patients (6, 7). Liu et al. showed that COVID-19 patients with thrombocytopenia had a higher mean platelet volume than those with a normal platelet count (26). Wool and Miller suggested that SARS-CoV-2 infection is associated with an increase in large immature platelets, as megakaryocytes respond to higher platelet consumption (27). Finally, patients with COVID-19 have increased number of immature platelets, which are known to be more active, even at normal platelet counts (28, 29). It is not yet clear whether this could be one explanation for increased thrombosis in COVID-19 patients. We have also observed that platelets from severely ill COVID-19 patients are larger in size and show balloon-like structures (unpublished observations). Platelets from COVID-19 patients are characterized by increased surface expression of glycoproteins (GPs), such as GPIIb/IIIa, which are responsible for cell adhesion and transduction of extracellular signals into the platelets (30).

### Immune-Mediated Platelet Activation in COVID-19

The association between the pathophysiology of COVID-19 and thrombosis seems to be multifactorial involving cellular and plasmatic components of the hemostatic system and players of the innate immune response to the infecting pathogen. The correlation between D-dimer levels and the surface expression of CD62P in severe COVID-19 patients suggests an association between platelet activation and COVID-19 associated coagulopathy (31). The mechanisms by which platelets are activated and express a procoagulant phenotype in COVID-19 are not clear. The release of a high amount of cytokines (cytokine storm) has been described in COVID-19 patients, which indicates a close correlation between ineffective immune responses to SARS-CoV-2, severe pneumonia, and life-threatening microangiopathy (32, 33). Moreover, enhanced production of C-reactive protein, interleukin-6 (IL-6), IL-8, and tumor necrosis factor alpha (TNF-alpha) during an immune response to systemic inflammation has been associated with increased thrombotic events (34). This might explain the thromboinflammation in severe COVID-19 infection.

Some evidence suggests a direct interaction between SARS-CoV-2 and platelets (12). Human platelets can express angiotensin-converting enzyme 2 (ACE2) and transmembrane protease serine 2 (TMPRSS2), which are responsible for the entry of SARS-CoV-2 into the cell (35). SARS-CoV-2 mRNA has been shown in platelets of some COVID-19 patients, suggesting infection of platelets with SARS-CoV-2 (36). Direct stimulation of platelets by SARS-CoV-2 and Spike protein enhances the release of coagulation factors, the secretion of inflammatory factors, and the formation of leukocyte–platelet aggregates (35). Koupenova et al. recently reported that SARS-CoV-2 can directly induce programmed cell death and extracellular vesicle release in platelets (12). It is important to note that not all studies demonstrated ACE2 expression on human platelets (36).

Platelets can also contribute to inflammation in COVID-19 (37). The levels of inflammatory cytokines such as IL-1beta, IL-18, sCD40L, and TxB_2_ were increased in blood and in platelets upon stimulation with thrombin in patients with COVID-19 infection (38). Increased levels of platelet-monocyte and platelet-granulocyte aggregates have been shown in patients with COVID-19 (37). Furthermore, platelets also contribute to high plasma levels of fibrinogen, von Willebrand factor, and factor XII in COVID-19 patients (37). Critically ill COVID-19 patients have a higher level of platelet-monocyte aggregates than those with mild/asymptomatic infection (31). Platelet/monocyte interactions lead to tissue factor expression on monocytes, which might contribute to hypercoagulability in critically ill COVID-19 patients (31). Platelets contribute not only to the hypercoagulable state in COVID-19 patients but also to the systemic inflammatory response (cytokine storm) by releasing inflammatory mediators and interacting with other immune cells (37, 38).

Another interesting area of research is the role of neutrophil extracellular traps (NETs) in COVID-19 induced thromboinflammation (39). NETs consist of nucleosomes and histone proteins. Upon activation, neutrophils generate NETs to capture and inactivate pathogens. Neutrophils of patients with COVID-19 release a higher amount of NETs (40). Platelet-neutrophil interactions through P-selectin/PSGL-1 can mediate NET formation (41). NETs can then trigger thrombosis by inducing tissue factor-mediated coagulation, platelet adhesion, and recruitment of platelet adhesive proteins (42).

Another mechanism of platelet involvement in COVID 19 is through antibody-mediated platelet activation. Nazy et al. suggested that platelets are the drivers of thromboembolic complications in COVID-19 infection (8). It has also been reported that platelets from severe COVID‐19 patients show a procoagulant phenotype, characterized by externalization of phosphatidylserine and release of CD62P (9). Additionally, platelets isolated from severe COVID-19 patients show increased calcium plasma concentration and inner-mitochondrial-transmembrane potential (Δψ) depolarization (9). Sera from COVID-19 patients activate platelets in serotonin release assay and this effect was completely inhibited by blocking FcyRIIa with mAb IV.3 (8). Most importantly, it has been reported that sera and IgG fractions from these patients are able to induce a procoagulant phenotype in platelets from healthy donors (9, 10) and are associated with phosphatidylserine externalization and P-selectin expression (11). On activated platelets, phosphatidylserine acts as a binding site for plasma coagulation factors and propagates actin and fibrin formation (43–45). Taken together these results indicate that one factor in the serum activates the inside-out signaling cascade and promotes the translocation of negatively charged phospholipids to the outer membrane leaflet. Our group recently showed that IgGs from severe COVID‐19 patients induce platelet activation through the PI3K/AKT signaling pathway *via* FcγRIIA (10). In particular, inhibition of FcγRIIA blocked AKT and PI3K phosphorylation preventing antibody-mediated platelet adhesion and thrombus formation in severe COVID-19 patients (10). More importantly, the inhibition of AKT and PI3K phosphorylation, with BAY1125976 or BYL719, prevented the generation of procoagulant platelets (10), which could be a promising therapeutic target in COVID-19 patients.

**Figure 1:**
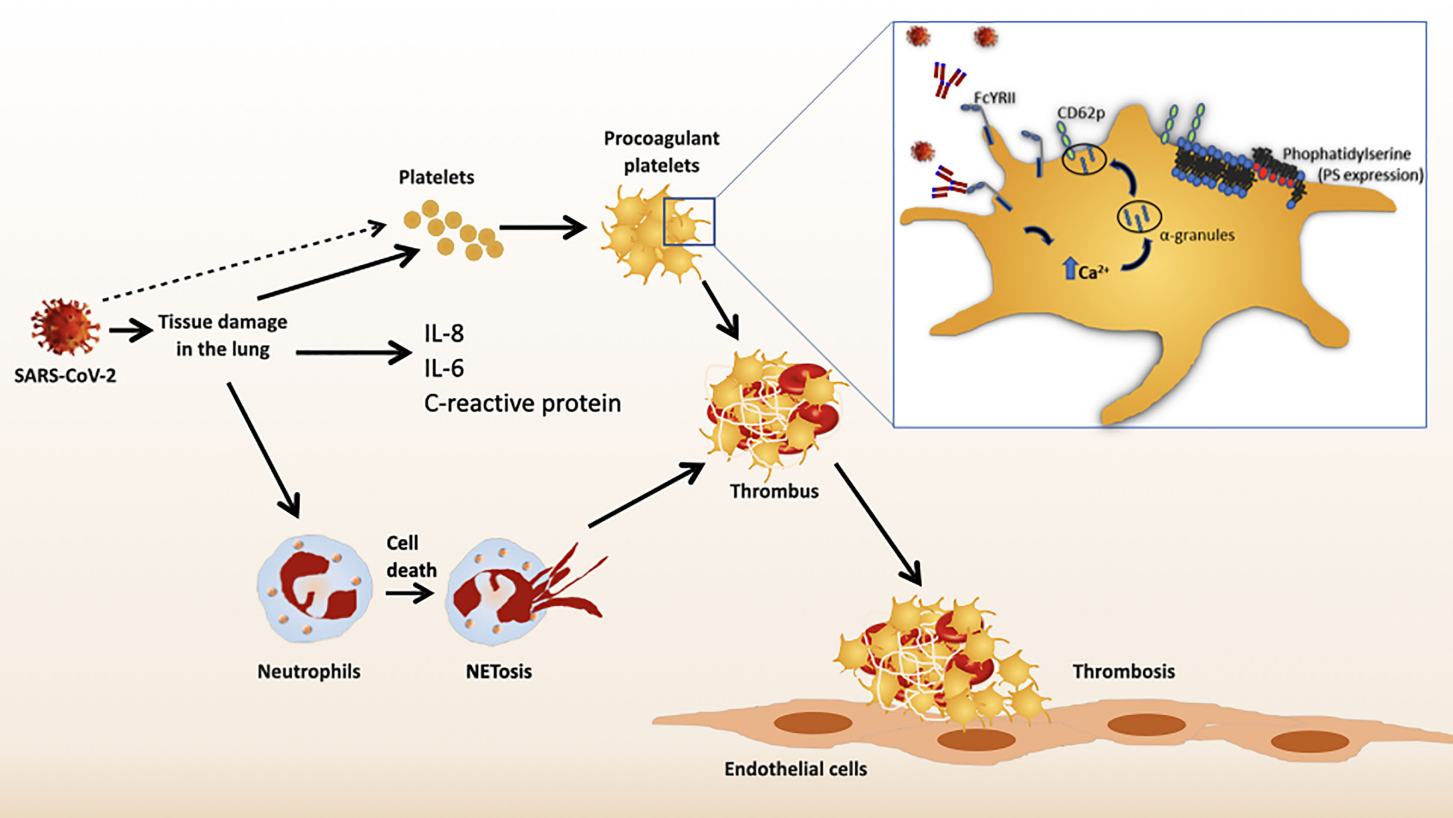
Schematic representation of immune mediated platelet activation in COVID-19.

The definitive characterization of these platelet-activating IgG antibodies has not been performed before. There is a correlation between increased neutralizing antibody titers against SARS-CoV-2 and the severity of COVID-19 infection (46). Most recently, Bye et al. showed that immune complexes containing recombinant SARS-CoV-2 spike protein and anti-spike IgG activate platelets only after aberrant glycosylation of the Fc domain (47). Compared to non-severe patients, patients in the intensive care unit had higher concentrations of afucosylated IgG antibodies against SARS-CoV-2 (48). Finally, immune complexes containing afucosylated IgG could activate platelet FcγRIIA. Clustering of FcγRIIA from platelets, induced by ligand binding, triggers intracellular signaling. Therefore, activation of FcγRIIA by afucosylated anti-spike IgG could further exacerbate thromboinflammation in COVID-19 patients (47). The proposed pathological mechanisms are summarized in **Figure 1**.

## Vaccine-Induced Immune Thrombotic Thrombocytopenia

VITT is an autoimmune condition characterized by thrombocytopenia and life-threatening thrombotic events in the arterial and venous circulation. Several major professional societies have published recommendations for the diagnosis and treatment of VITT (49). Although the published guidelines have slight differences in diagnostic criteria for VITT, they mainly include the following: clinical diagnostic criteria include vaccination with a vector-based COVID-19 vaccine (AstraZeneca and Johnson&Johnson/Jannsen) 4 to 30 days previously, venous or arterial thrombosis, and thrombocytopenia. Serological confirmation includes PF4 antibody ELISA and confirmatory PF4 platelet activation assay (heparin-induced platelet activation assay, serotonin release assay, or P-selectin expression assay.

### Incidence and Risk Factors

The true incidence of VITT is not known. As of 31 July 2021, a total of 1503 cases of thrombosis with thrombocytopenia have been reported worldwide after 592 million doses of ChadOx1 nCov19, suggesting a rate of one case per 394 000 vaccinations (50). See et al. recently reported an incidence of one case per 282 000 vaccinations with Ad26.Cov2.S (51).

Initial case reports defined women under 40 years of age as the risk group, but the reason for this finding was that ChadOx1 nCov19 was first available for medical personnel under 60 years of age in Europe, who are predominantly female (13, 14, 16). However, later reports with larger case numbers showed that there was not much difference between genders and age groups in VITT (52, 53).

### Clinical Presentation

Initial physical signs appear in most cases within 2 weeks after vaccination. Due to delays in recognizing symptoms or seeking medical attention, patients may be seen at a medical facility 4 weeks or more after vaccination. The symptoms reflect thrombocytopenia and the location of the thrombosis. Patients with severe thrombocytopenia develop petechiae, bruising, or even hematoma. More than half of the patients with severe thrombocytopenia have cerebral venous sinus thrombosis (52, 54). Severe headache is the initial symptom in patients with cerebral venous sinus thrombosis (54). Altered mental status or focal neurological deficit may indicate the presence of cerebral ischemia and hemorrhage. One-third of the patients with cerebral venous sinus thrombosis have intracranial hemorrhage at presentation (55). The presence of abdominal pain at presentation suggests thrombosis of the splanchnic veins. Patients with pulmonary arterial embolism have shortness of breath and chest pain, and those with deep vein thrombosis of the lower extremities have leg pain or leg swelling.

The mortality rate was up to 60% in the initial case series (13, 14). With better recognition of the syndrome and increased awareness among medical professionals, the mortality rate has decreased significantly (55). The reported mortality decreased from 47% to 22% after 28 March 2021 in cases with VITT and cerebral venous sinus thrombosis (55). Pavord reported a mortality rate of 22% in a recent case series of 231 patients (52).

### Pathophysiology

VITT is an autoimmune condition characterized by antibodies able to activate platelets, triggering subsequent life-threatening thrombotic events in the arterial and venous circulation. Individuals with VITT have been shown to develop severe coagulopathy, thrombocytopenia, and an elevated D-dimer concentration. Earlier reports described that patients with VITT contain high titer antibodies directed against PF-4 (13–16). PF4 is a molecule stored in the α granules of platelets and released during platelet activation. In VITT, these high-titer pathologic anti-PF4 antibodies bind with the FcγRIIA receptor on platelets. This in turn activates intracellular signaling leading to platelet activation and PF4 antibody clustering on the surface of activated platelets (56).

A further study hypothesized that FcγRIIA-mediated binding of PF4 antibodies with platelets and monocytes might cause monocytic activation and simultaneous release of procoagulant platelet microparticles (MPs). The potential expression of tissue factor on these procoagulant MPs might be the reason behind the higher thrombogenicity of the cerebral microvascular compartment (57). The pathophysiology of VITT has been proposed to closely resemble that of autoimmune heparin-induced thrombocytopenia (aHIT), also caused by anti-PF4 antibodies. In classic HIT, anti PF4 antibodies recognize the ionic complex of positively charged PF4 and negatively charged heparin (58). In contrast, anti-PF4 antibodies in aHITT do not require heparin for their binding and platelet activation (59).

Autoantibodies produced in HIT induce a broad spectrum of immune activation, especially Fcγ receptor-dependent stimulation of immune cells, such as monocytes or granulocytes, and subsequently enhanced thrombin generation. Neutrophil extracellular traps (NETs) induced by HIT antibodies also play a significant role in HIT pathogenesis (60). It has also been proposed that neutrophil activation and NETs are also involved in VITT pathophysiology (61). Holm et al. showed neutrophil accumulation, IgG and NETs in a thrombus from a patient with VITT (62).

Antibody binding to FcγRIIA also plays a significant role in thrombocytopenia and enhanced platelet clearance in HIT (63). Current evidence suggests that VITT antibodies might have a similar mechanism leading to increased thrombosis and thrombocytopenia (16, 64). Although HIT is usually triggered by heparin exposure, aHIT is reported to develop without any prior exposure to heparin (65). Despite various pathophysiological and clinical similarities reported between VITT and aHIT, a scanning mutagenesis analysis recently described a distinct binding pattern between VITT and HIT antibodies. Unlike HIT, anti-PF4 antibodies VITT patients were shown to bind to eight surface amino acids, which were all located within the heparin-binding site of PF4 (66).

Since adenoviral vector vaccines have been found to be the main trigger behind VITT, it is still uncertain how these vaccines trigger this syndrome. Recent studies hint toward a direct interaction between adenoviral vectors and PF4, triggering an anti-PF4 response and subsequent thromboinflammation. Using super-resolution microscopy and transmission electron microscopy, vaccine components were visualized by forming antigenic complexes with PF4 on platelet surfaces, and anti-PF4 antibodies obtained from VITT patients bound to these complexes (56). The addition of DNA leads to an enhanced PF4/vaccine complex formation. In a recent study Baker and colleagues, demonstrated that all three adenoviruses deployed as vaccination vectors bind to PF4. An electrostatic interaction mechanism between PF4 and ChAdOx1 nCov19 viral vector was determined using computational simulations, and later confirmed experimentally by surface plasmon resonance. This study hints toward the direct involvement of vector adenovirus, forming stable complexes with PF4 and subsequent pathogenesis in VITT (67). Henceforth the binding of PF4 with the viral capsid might create a novel antigen, which is subsequently taken up by monocytes and trafficked to lymph nodes, where it stimulates proliferation of anti-PF4 memory B cells. (56, 68) Vaccine components, such as EDTA (edetic acid) or other human proteins in the vaccine, might also contribute to promoting pro-inflammatory signaling pathways that lead to a hyperactive activated immune response. Recently, Ostrowski et al. showed that after vaccination biomarkers of inflammation and platelet activation increased (69). Furthermore, thrombin generation is enhanced after vaccination with ChadOx1 nCov19 but not after vaccination with the mRNA vaccine, BNT162b2 (69).

**Figure 2:**
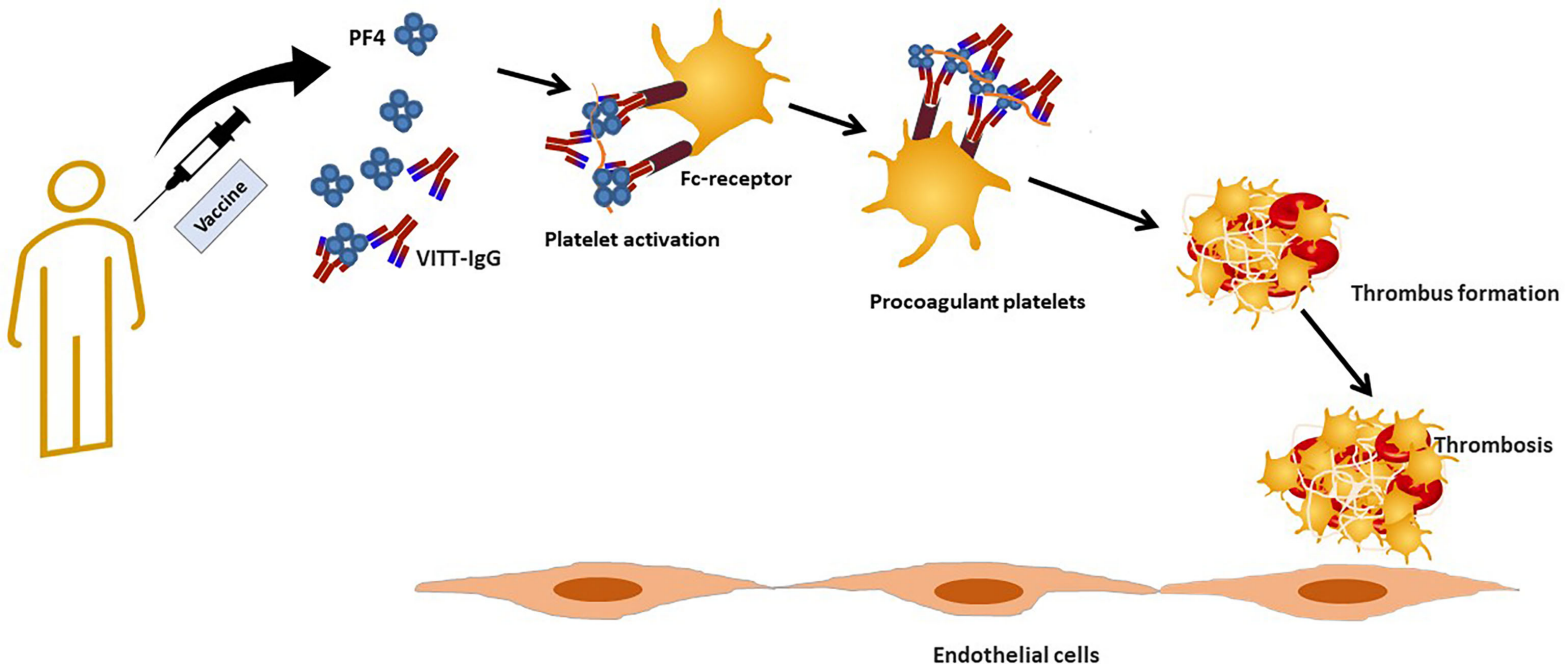
Proposed pathologic events in vaccine induced immune thrombotic thrombocytopenia.

Finally, it was hypothesized that there is a cross-reactivity between the spike protein and PF4 in patients with VITT. We have shown that there is no correlation between anti-SARS-CoV-2 antibodies and anti-PF4 antibodies (70). Anti-PF4 antibodies purified from VITT patients do not cross react with SARS-CoV-2 spike protein *in vivo* (71). However, a recent study showed that anti-RBD antibodies produced after vaccination do not show significant binding to PF4 (72), whereas purified polyclonal PF4 antibodies bind to Spike-RBD (72). The discrepancy between these studies may be because the earlier study (71) used anti-PF4 antibodies that had been purified from VITT patients, so they were bound in complexes and therefore could not be purified or bind to other molecules. The authors suggest that RBD and PF4 may form complexes similar to heparin-PF4 that can be recognized by either anti-RBD or anti-PF4 antibodies (72).The proposed pathological mechanisms are summarized in **Figure 2**.

## Future Therapeutic Perspectives

Several clinical trials have been initiated to evaluate the effects of antiplatelet drugs in patients with COVID-19. A meta-analysis showed a reduction in mortality in COVID-19 patients treated with aspirin (73). A recent multinational observational study reported that anti-platelet therapy (aspirin as monotherapy in 88% of the patients) is associated with a reduced mortality risk in multivariable analyses (74). On the other hand, in a large randomized, controlled, open-label study (RECOVERY), aspirin failed to reduce the 28-day mortality rate or need for mechanical ventilation in hospitalized COVID-19 patients (75). Furthermore, major bleeding events were higher in aspirin treated COVID-19 patients (75).

Platelet activation *via* FcγIIA is the central mechanism in VITT (10). Intravenous IVIG can interfere in this step and “cool down” VITT (76). Antiplatelet drugs (indomethacin, ticagrelor) and tyrosine kinase inhibitors also block platelet activation induced by plasma from VITT patients *in vitro* (77). However, further studies are needed to evaluate their therapeutic potential before they are used in patients.

## Conclusions

COVID-19 continues to challenge us on several fronts. Accumulating evidence proved that platelets play an important role in COVID-19 pathophysiology by either inducing thrombosis or interacting with other immune cells to amplify thromboinflammation. Antiplatelet drugs are not routinely used in COVID-19 patients. The results of the recently published studies evaluating the effects of antiplatelet drugs in patients with COVID-19 are contradictory. Further studies are needed to better define the role of platelets in COVID-19 and to evaluate the use of therapies targeting platelets.

As the vaccination campaign to contain the pandemic will continue, there will certainly be new cases of VITT, and medical professionals around the world will be looking for answers on how to deal with such cases. A better understanding of the pathophysiology of VITT will help us develop efficient treatment strategies.

## Author Contributions

GU, AS, LP, and TB wrote the manuscript. GU, AS, and LP performed the literature review and data collection. AS and LP designed the Figure. GU and TB revised the manuscript. All authors read and approved the manuscript.

## Funding

This work was supported by grants from the German Research Foundation and from the Herzstiftung to TB (BA5158/4 and TSG-Study). We acknowledge support from the Open Access Publishing Fund of University of Tübingen.

## Conflict of Interest

TB has received research funding from CoaChrom Diagnostica GmbH, DFG, Robert Bosch GmbH, Stiftung Transfusionsmedizin und Immunhämatologie e.V.: Ergomed, Surrey, DRK Blutspendedienst, Deutsche Herzstiftung, Ministerium fuer Wissenschaft, Forschung und Kunst Baden-Wuerttemberg, has received lecture honoraria from Aspen Germany GmbH, Bayer Vital GmbH, Bristol-Myers Squibb GmbH & Co., Doctrina Med AG, Meet The Experts Academy UG, Schoechl medical education GmbH, Mattsee, Stago GmbH, Mitsubishi Tanabe Pharma GmbH, Novo Nordisk Pharma GmbH, has provided consulting services to: Terumo, has provided expert witness testimony relating to heparin induced thrombocytopenia (HIT) and non‐HIT thrombocytopenic and coagulopathic disorders. All of these are outside the current work.

The remaining authors declare that the research was conducted in the absence of any commercial or financial relationships that could be construed as a potential conflict of interest.

## Publisher’s Note

All claims expressed in this article are solely those of the authors and do not necessarily represent those of their affiliated organizations, or those of the publisher, the editors and the reviewers. Any product that may be evaluated in this article, or claim that may be made by its manufacturer, is not guaranteed or endorsed by the publisher.
